# Dataset of observables for UOX and MOX spent fuel extracted from Serpent2 fuel depletion calculations for PWRs

**DOI:** 10.1016/j.dib.2023.109412

**Published:** 2023-07-13

**Authors:** Victor J. Casas-Molina, Augusto Hernandez-Solis, Pablo Romojaro, Ivan Merino-Rodríguez, Nerea Aguilera-Gómez

**Affiliations:** a Belgian Nuclear Research Centre (SCK CEN), Boeretang 200, 2400 Mol, Belgium; b Universidad Politécnica de Madrid (UPM), José Gutiérrez Abascal, 2, 28006 Madrid, Spain; c Universidad Católica del Maule (UCM), Talca 3460000, Chile

**Keywords:** Spent nuclear fuel, UOX, MOX, Nuclide inventory, Decay heat, Neutron and gamma emission, Nuclear safeguards, Radiotoxicity, Serpent2, Fuel depletion calculations

## Abstract

This database contains the isotopic mass density and the contribution to activity, decay heat, photon emission, spontaneous fission rate, (α,n) emission rates and radiotoxicity of 150 nuclides that are present in nuclear fuel irradiated in PWRs. These nuclides are of paramount importance for nuclear waste characterization and fuel cycle analysis. These values were obtained by depletion calculations based on a 3D pin-cell geometry model and performed with the Monte Carlo reactor physics burnup calculation code Serpent2, with state-of-the-art nuclear data libraries and relevant methods. The calculations cover a wide range of burnup levels for conventional PWRs and take into account both UOX and MOX fuel. A broad span for initial enrichment for UOX (from 1.5% to 6.0%), and for both the initial plutonium content (from 4.0% to 12.0% and the plutonium isotopic composition of MOX has been considered. This database has been made publicly available due to its relevance in the fields of waste and fuel characterization, nuclear safeguards and radiation protection, and it will allow other potential users to avoid the time-consuming calculations required to obtain the aforementioned data. Additionally, it constitutes an interesting dataset for model training in machine learning applications related to nuclear science and engineering.

**Specifications Table**

**Table:** 

Subject	Nuclear Energy and Engineering
Specific subject area	Nuclear spent fuel characterization, nuclear safeguards and fuel cycle analysis.
Type of data	Table
How the data were acquired	Fuel depletion calculations with a representative PWR pin-cell model using the Sepent2 Monte Carlo Continuous-energy code, version 2.2.0
Data format	Raw Filtered
Description of data collection	Data were extracted from the depletion output files from Serpent2 calculations with SerpentTools and then processed in Python using Pandas.
Data source location	• Institution: Belgian Nuclear Research Centre (SCK CEN) • City: Mol • Country: Belgium
Data accessibility	Repository name: Mendeley Data Data identification number: DOI: 10.17632/shv89y2zzd.5 Direct URL to data: https://data.mendeley.com/datasets/shv89y2zzd/5

## Value of the Data

• The present database contains information about uranium oxide (UOX) and mixed oxide (MOX) fuel, both irradiated in pressurized water reactors (PWR), i.e., the time evolution of 150 isotopes which have been selected for their utmost importance related to spent nuclear fuel characterization (burnup indicators, gamma emitters), fuel cycle analysis (heavy metal and fission product masses, radiotoxicity) and nuclear safeguards (neutron emitters and decay heat contributors).
• Additionally, decay heat, radiotoxicity, activity, spontaneous fission neutron emission rates, (α,n) emission rates and photon emission rates are present in the dataset for each specified isotope, which supplements the nuclide inventory with ready-to-use information for many fields of research in which these observables could be involved.
• The dataset can contribute to the research on nuclear safeguards, nuclear medicine, nuclear waste, spent fuel characterization and similar fields, allowing to avoid the time and resource consuming calculations that are required with fuel depletion codes.
• Provided data are also relevant for Deep Learning and Machine Learning models, gamma spectroscopy studies and educational purposes, both for data science and nuclear science and technology.
• The present dataset complements the efforts of previous published libraries in Refs. [1,2], in which similar datasets obtained with SCALE6.1 [3] and Serpent2 [4] codes were published.
• The present data provide novel information on the above-mentioned observables for each nuclide, generated with consistent state-of-the-art nuclear data (ENDF/B-VIII.0 nuclear data library [5]).

## 1 Objective

This library consists of relevant data for the nuclear technology field that were derived from Serpent2 depletion calculations. Those calculations were performed aiming to generate a database to train a Deep Learning model capable of predicting the final isotopic inventory for PWR spent nuclear fuel (UOX and/or MOX) employing as general inputs, the initial fuel composition (enrichment or plutonium content) and the target discharge burnup [6]. The model is part of the irradiation module of ANICCA [7], the in-house fuel cycle analysis tool from SCK CEN.

## 2 Data Description

The dataset consists of two comma separated value files (.csv), one for each type of fuel. The first file, *SCKCEN_UOX_PWR.csv* contains the information related to UOX fuel and it is structured in a matrix conformed by 63 531 (rows) x 1058 (columns). The first column is ‘BU’ and indicates the burnup in MWd/kg_HM_; the second column is named ‘IE’ and its values indicate the initial enrichment for UOX fuel in percentage. The next 150 columns contain the mass density in g/cm^3^ of the nuclides sorted by the atomic mass that were selected based on Refs. [8] and [9]. Additionally, some other nuclides have been included to ensure mass conservation:

‘*H3*’, ‘*He4*’, ‘*O16*’, ‘*Se79*’, ‘Se80’, ‘Se82’, ‘Br81’, ‘Kr81’, ‘Kr83’, ‘Kr84’, ‘Kr85’, ‘Kr86’, ‘Rb85’, ‘Rb87’, ‘Sr88’, ‘***Sr90***’, ‘Y89’, ‘***Y90***’, ‘Zr90’, ‘Zr91’, ‘Zr92’, ‘*Zr93*’, ‘Zr94’, ‘Zr96’, ‘Nb93m’, ‘Mo95’, ‘Mo96’, ‘Mo97’, ‘Mo98’, ‘Mo100’, ‘Tc99’, ‘Ru100’, ‘Ru101’, ‘Ru102’, ‘Ru104’, ‘***Ru106***’, ‘Rh103’, ‘**Rh106**’, ‘Pd104’, ‘Pd105’, ‘Pd106’, ‘Pd107’, ‘Pd108’, ‘Pd110’, ‘Ag109’, ‘Cd110’, ‘Cd111’, ‘Cd112’, ‘Cd113m‘, ‘Cd114’, ‘Cd116’, ‘Sn116’, ‘Sn117’, ‘Sn118’, ‘Sn119’, ‘Sn120’, ‘Sn122’, ‘Sn124’, ‘*Sn126*’, ‘Sb121’, ‘Sb123’, ‘*Sb125’*, ‘*Te125*’, ‘Te125m’, ‘Te128’, ‘Te130’, ‘I127’, ‘I129’, ‘Xe128’, ‘Xe130’, ‘Xe131’, ‘Xe132’, ‘*Xe134*’, ‘Xe136’, ‘**Cs133**’, ‘**Cs134**’, ‘Cs135’, ‘***Cs137***’, ‘Ba134’, ‘Ba136’, ‘Ba137’, ‘**Ba137m**’, ‘Ba138’, ‘La139’, ‘*Ce140*’, ‘Ce142’, ‘**Ce144**’, ‘Pr141’, ‘Nd142’, ‘Nd143’, ‘Nd144’, ‘Nd145’, *‘Nd146*’, ‘**Nd148**’, ‘Nd150’, ‘Pm147’, ‘Sm146’, ‘Sm147’, ‘Sm148’, ‘**Sm149**’, ‘Sm150’, ‘Sm151’, ‘Sm152’, ‘Sm154’, ‘Eu153’, ‘**Eu154**’, ‘Eu155’, ‘Gd156’, ‘Gd158’, ‘Tb159’, ‘Ho166m’, ‘*Pb206*’, ‘*Pb207*’, ‘*Pb208*’, ‘*Pb210*’, ‘*Bi209*’, ‘Ac227’, ‘Th228’, ‘Th229’, ‘Th230’, ‘Th232’, ‘Pa231’, ‘*U232*’, ‘*U233*’, ‘*U234*’, ‘*U235*’, ‘*U236*’, ‘*U238*’, ‘*Np237*’, ‘***Pu238***’, ‘***Pu239***’, ‘***Pu240***’, ‘***Pu241***’, ‘*Pu242*’, ‘*Pu244*’, ‘***Am241***’, ‘*Am242m*’, ‘*Am243*’, ‘*Cm242*’, ‘*Cm243*’, ‘***Cm244***’, ‘*Cm245*’, ‘*Cm246*’, ‘*Cm247*’, ‘*Cm248’*, ‘*Cm250*’, ‘*Bk249*’, ‘*Cf249’*, ‘*Cf250*’, ‘*Cf251*’.

The isotopes are named by their chemical symbol and atomic mass. If an ‘m’ exists after the atomic mass, it means that the mentioned isotope is in a metastable state. If in the above list an isotope appears in bold, it is because it has been obtained from [8]; if it is in italic, it has been extracted from [9] and if it is in both ways it is because it appears in both Refs. The rest are the contributors added for mass conservation.

The remaining columns have the same structure and order as the columns containing the mass density with the addition of a suffix to their labels, the only exception is for the columns of (α,n) emission rate, that only exist for the isotopes Pu238, Pu239, Pu240, Am241, Cm242 and Cm244. Information about the meaning of suffixes is given in Table 1, being the order of appearance for the suffixes in the table the same as the columns order in the dataset.

**Table 1:** Suffixes employed in the dataset. Example given for Pu239.

‘Pu239_A’	Activity in Becquerels of Pu239
‘Pu239_H’	Decay Heat in Watts of Pu239
‘Pu239_SF’	Spontaneous fission rate in fissions per second of Pu239
‘Pu239_AN’	(α,n) emission rate in neutrons per second of Pu239
‘Pu239_GSRC’	Photon emission rate in photons per second Pu239
‘Pu239_ING_TOX’	Ingestion toxicity in Sieverts of Pu239
‘Pu239_INH_TOX’	Inhalation toxicity in Sieverts of Pu239

The second file, named as *SCKCEN_MOX_PWR.csv,* contains the information related to MOX fuel and it is structured in a 877 500 rows x 1064 columns matrix. The first six columns are: ‘Pu238_IPC’, ‘Pu239_IPC’, ‘Pu240_IPC’, ‘Pu241_IPC’, ‘Pu242_IPC’, ‘Am241_IPC’ and indicate the composition of the initial plutonium vector for the fresh MOX fuel in percentage, i.e., the proportion of the isotopes present in the initial fuel composition. The seventh column is ‘BU’ and indicates the same as in the UOX dataset. The eighth column is ‘IPC’ and indicates the initial plutonium composition for the fresh fuel in percentage taken over the heavy metal mass. The rest of columns are ordered and named with the same shape and format that the one used for the UOX file. ‘BU’, and ‘IE/IPC’ columns store the samples generated to cover the ranges with the steps and limits described in the *Methods* section of this document. As can be noticed, MOX dataset is larger due to the larger sample space generated for the variation of the Pu vector, as described in the *Methods* sections.

When dealing with MOX fuel data, it is strongly recommended to either open the .csv file in chunks or filter the data beforehand by selectively removing those values in the index (or columns) that are not of interest. For instance, the unpacking operator can be employed in Pandas for selecting a desired range of columns to be loaded:

import pandas as pd

data_features=pd.read_csv(‘SCKCEN_MOX_PWR.csv’, usecols=[*range(8)])

data_targets=pd.read_csv(‘SCKCEN_MOX_PWR.csv’, usecols=[*range(8,163)])

On the other hand, for the UOX dataset, one could use:

import pandas as pd

data=pd.read_csv(‘SCKCEN_UOX_PWR.csv’,usecols=[*range(152)])

data_features, data_targets = data.values[:,0:2],data.values[:,2:152]

Additionally, the dataset can also be filtered according to the names of the columns, for instance, if only the spontaneous fission rates are needed, they can be included in a smaller dataset by doing:

data_targets_filtered=data_targets.filter(regex=‘_SF$’, axis=1)

## 3 Experimental Design, Materials and Methods

### 3.1 Methods

The UOX fuel initial isotopic vector consists of U234, U235, U238. The weight fraction of said isotopes can be expressed as a function of the initial enrichment IE in percentage. The expression for obtaining $w_{234}$ has been obtained from [10]:

(1) $$\begin{array}{c} w_{235}=\mathrm{IE}/100 \end{array}$$

(2) $$w_{234}=\left(1.036\cdot IE-0.449\right)\cdot 10^{-4}$$

(3) $$w_{238}=1-w_{235}-\;w_{234}$$

The MOX initial plutonium fuel vectors were generated following correlations for Reactor-Grade plutonium present in MOX fuel available in [11]. Said correlations allow calculating the remaining isotopes of Pu from the Pu239 concentration. The proportion of Pu239 was randomly sampled from a normal distribution with mean ∼52.8% and a standard deviation of ∼10.6%. These parameters were set according to the sample extracted from [12]. The Am241 proportion was sampled from a random distribution that removes any value from 0 to 100% from the Pu241 total proportion derived from the previous sampling and converts it to Am241, in an attempt to simulate the natural decay of Pu241 to Am241 over the time.

**Fig. 1:**
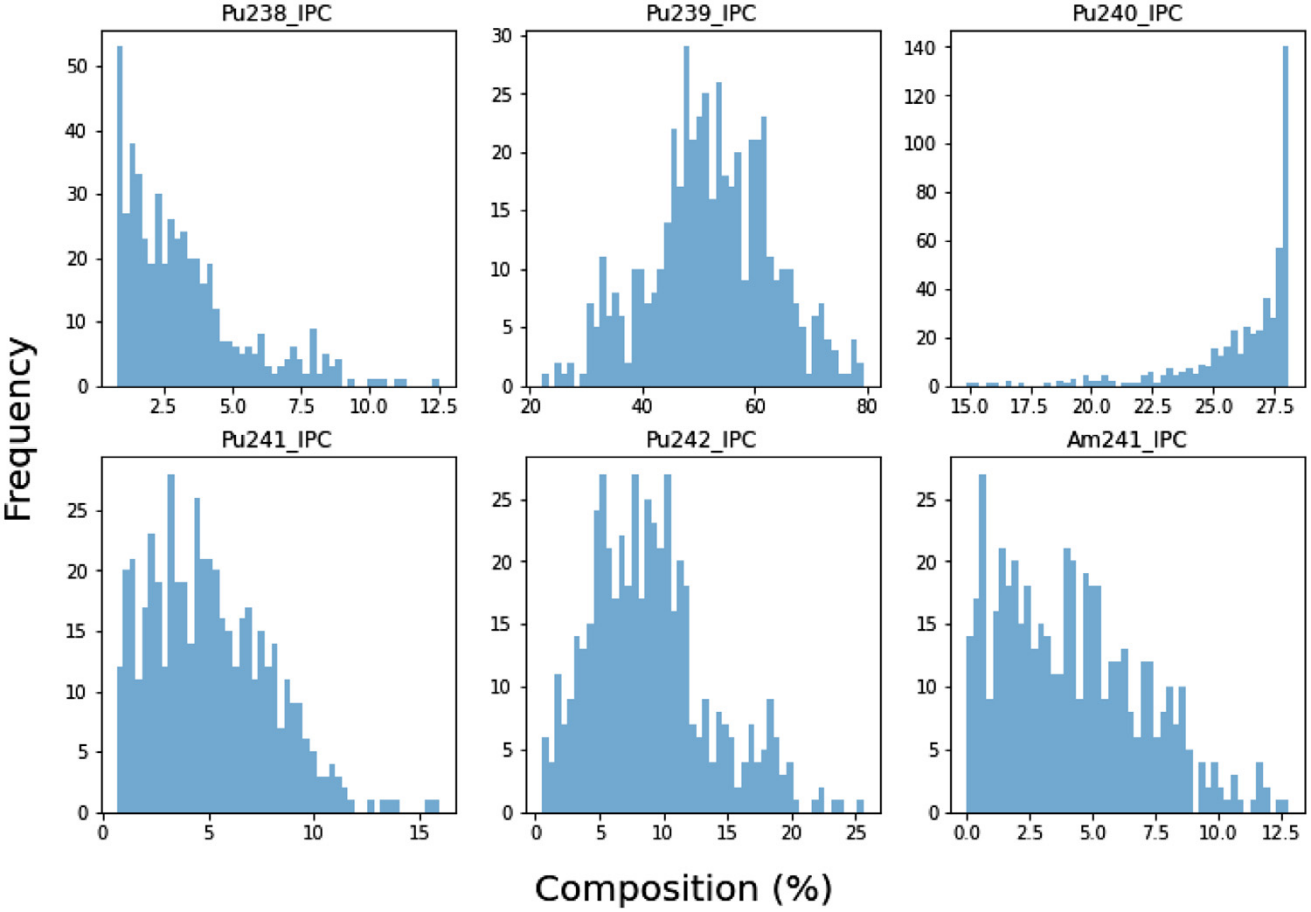
Histograms of the samples (i.e. first six columns of MOX dataset) produced by combination of the correlations and the random sampling of a normal distribution for the Pu239.

Due to computational limitations, only 500 samples were extracted for the Pu vector (for each five Pu enrichments). A detailed overview of the distributions for the various isotopes can be found in Fig. 1.

In both types of fuel, O16 has been added subsequently to achieve a 2:1 atomic ratio of uranium and plutonium dioxide. Finally, the mass percentages have been normalized.

To sample the eight-dimensional (8D) space generated by the variables of the initial MOX vector, namely Pu238_IPC, Pu239_IPC, Pu240_IPC, Pu241_IPC, Pu_242_IPC and Am241_IPC plus IPC, and BU, a regular grid pattern was used. This approach resulted in the generation of 500 variations of the initial MOX vector for five equally spaced IPC values, resulting in a total of 2 500 sampled initial isotopic compositions. These compositions were then explored across the entire burnup range, ranging from 0 to 70 MWd/kg_HM_ in increments of 0.2. Consequently, the MOX database contains 875 500 rows, representing 2 500 initial isotopic vectors per 351 burnup steps.

Similarly, the UOX database derives from a simpler two-dimensional (2D) space. It consists of these aforementioned 351 burnup steps, repeated for 181 compositions ranging from 1.5% to 6% enrichment in increments of 0.025%. This results in a total of 63 531 rows in the UOX database

Neutron emission rate (α,n) term, $s_{\left(\alpha ,n\right)}\left(t\right)$, was subsequently calculated using the Thick Target Yield approach:

(4) $$s_{\left(\alpha ,n\right)}\left(t\right)=\sum_{i}N_{i}\left(t\right)\;\lambda_{\alpha ,i}\sum_{j,k}P_{i}\left(E_{\alpha ,k}\right)Y_{l}\left(E_{\alpha ,k}\right)$$

where $N_{i}\left(t\right)$ is the nuclide i number density, $\lambda_{\alpha ,i}$ is the decay constant for α-decay of nuclide i, $P_{i}\left(E_{\alpha ,k}\right)$ is the probability for emission of an α-particle with energy $E_{\alpha ,k}$ from nuclide i undergoing α-decay and $Y_{l}\left(E_{\alpha ,k}\right)$ is the neutron yield for an α-particle with energy $E_{\alpha ,k}$ in the target material l. The quantities $\lambda_{\alpha ,i}$ and $P_{i}\left(E_{\alpha ,k}\right)$ were obtained directly from the nuclear data libraries. The determination of thick target yields $Y_{l}\left(E_{\alpha ,k}\right)$ requires calculation of (α,n) reaction rate during α-particle slowing down in the target material. In this case, the $Y_{l}\left(E_{\alpha ,k}\right)$  values were adopted based on the models available in the SCALE Code System [13].

### 3.2 Materials

Depletion calculations in Serpent2 are performed by successive runs of inputs with the desired material compositions and parameters setup for the combinations of composition and burnup depicted in the previous section. The geometry of the model is based on a cuboid containing a segment of a fuel rod, which is comprising both the fuel pellet and the cladding. This model, based on specifications from Ref. [14], is representative of PWR fuel. The simplified PWR pin-model employed is taking then the different range of compositions for UOX and MOX as the input of the material fuel definition (*mat fuel*), the burnup is set for a constant power according to the normalization of the total power of the reactor for that given volume. The parameters related to the general definition of the geometry and the input can be found in Table 2:

**Table 2:** General information about the Serpent2 model:

Serpent Version	*2.2.0*	
Enrichment	From 1.5% to 6.0% for UOX in steps of 0.025% From 4.0% to 12.0% for MOX, in steps of 2%	
Burnup	0 to 70 MWd/kg_HM_ in steps of 0.2 MWd/kg_HM_.	
Neutron Cross Section Library	ENDF/B-VIII.0	
Branching ratio libraries	ENDF/B-VIII.0	
Decay and fission yield data libraries	ENDF/B-VIII.0	
Time integration method	Constant extrapolation (*set pcc 0*)	
Power	161.9 W (*set power*)	
***Population***	(*set pop 5000 100 10*)	
Number of histories per generation	5000	
Number of active generations	100	
Number of inactive generations	10	
Boundary conditions	Reflective (*set bc 2*)	
***Geometry definition of the problem***	Cuboid 1.264916 cm x 1.264916 cm x 1.00 cm (L x W x H)	
	*pin*	*p1*
	*Fuel*	*0.41266*
	*clad* *water*	*0.474364*
	*surf s1 cuboid -0.632458 0.632458 -0.632458 0.632458 -0.5 0.5* *cell c1 0 fill p1 -s1* *cell c2 0 outside s1*	
Fuel pellet radius (cm)	0.412660	
Cladding radius (cm)	0.474364	
Fuel volume (cm^3^)	0.5349763956	
***Temperature***		
Fuel temperature (K)	900.0	
Cladding temperature(K)	600.0	
Water temperature (K)	600.0	
***Density***		
Fuel density (g/cm^3^)	10.07	
Cladding density (g/cm^3^)	6.49012	
Water density (g/cm^3^)	0.7245	

Once the calculations were completed, the Depletion Reader module from SerpentTools [15] was used to extract all the relevant information from the outputs stored in the database. The objects extracted from the Depletion Reader were parsed into a data frame using the Pandas tool, and then exported in comma-separated values format (csv).

## Ethics Statement

We, the authors, unequivocally state that we do not have any financial or monetary interests that could potentially influence the outcomes, conclusions, or interpretations presented in this work. Furthermore, we affirm that no human or animal experiments were conducted for the generation of the data utilized in this research.

## CRediT authorship contribution statement

**Victor J. Casas-Molina:** Investigation, Methodology, Software, Formal analysis, Data curation, Writing – original draft. **Augusto Hernandez-Solis:** Conceptualization, Supervision, Resources, Writing – review & editing. **Pablo Romojaro:** Project administration, Supervision, Validation, Resources, Writing – review & editing. **Ivan Merino-Rodríguez:** Writing – review & editing. **Nerea Aguilera-Gómez:** Software, Writing – review & editing.

## Declaration of Competing Interest

The authors declare that they have no known competing financial interests or personal relationships that could have appeared to influence the work reported in this paper.

## Data Availability

Dataset of observables for UOX and MOX spent fuel extracted from Serpent2 fuel depletion calculations for Pressurized Water Reactors (Original data) (Mendeley Data).
